# Towards 4-dimensional atomic force spectroscopy using the spectral inversion method

**DOI:** 10.3762/bjnano.4.10

**Published:** 2013-02-07

**Authors:** Jeffrey C Williams, Santiago D Solares

**Affiliations:** 1 Department of Mechanical Engineering, University of Maryland, College Park, MD 20742, USA

**Keywords:** atomic force microscopy, spectral inversion, spectroscopy, torsional harmonic cantilever, viscoelasticity

## Abstract

We introduce a novel and potentially powerful, yet relatively simple extension of the spectral inversion method, which offers the possibility of carrying out 4-dimensional (4D) atomic force spectroscopy. With the extended spectral inversion method it is theoretically possible to measure the tip–sample forces as a function of the three Cartesian coordinates in the scanning volume (*x*, *y* and *z*) and the vertical velocity of the tip, through a single 2-dimensional (2D) surface scan. Although signal-to-noise ratio limitations can currently prevent the accurate experimental implementation of the 4D method, and the extraction of rate-dependent material properties from the force maps is a formidable challenge, the spectral inversion method is a promising approach due to its dynamic nature, robustness, relative simplicity and previous successes.

## Introduction

Besides topographical imaging, a popular application of atomic force microscopy (AFM) is the measurement of probe–sample interaction force curves (force spectroscopy), generally based on contact and frequency-modulation methods [1–6]. The procedure is generally time-consuming because the acquisition of the force curve for each (*x*,*y*) location on the surface requires that the cantilever approaches and retracts from the sample at relatively low vertical speed, without traveling horizontally. If one wishes to fully characterize a 2D sample, one must slowly acquire force curves throughout the surface, one location at a time. In 2002, Stark et al. [7] introduced an AFM method for performing real-time spectroscopy simultaneously with topographic imaging (that is, while the cantilever images the surface at typical scan speeds), through acquisition and inversion of the spectral response of the tip motion. This approach permits extraction of the tip–sample interaction force as a component of the driving force acting on the cantilever and was demonstrated with standard cantilevers, although the low signal-to-noise ratio of certain regions of the spectrum limited its accuracy. In 2007, Sahin and co-workers [8] introduced a T-shaped cantilever with an off-centered tip located on one of the arms of the “T”, the so-called *torsional harmonic cantilever* (THC), on which the tip–sample interaction generates a torsional oscillation whose amplitude is enhanced by the soft and highly detectable fundamental torsional frequency. Such enhancement provided a more accurate means to implement the method of Stark et al. [7] and the improved technique has been validated experimentally [8–10], studied computationally [11–12] and also commercialized [Harmonix^TM^, Bruker Corporation (formerly Veeco Instruments)].

**Figure 1:**
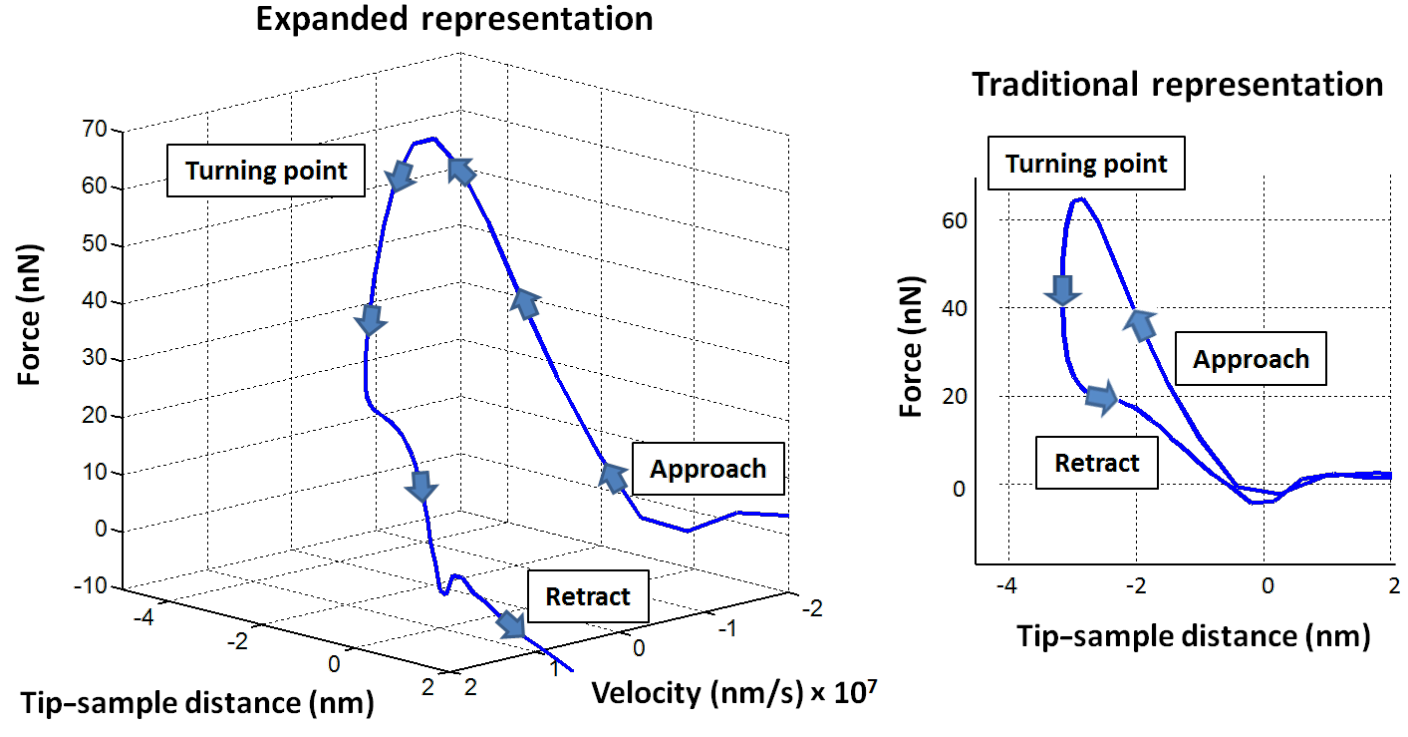
Simulated reconstructed tip–sample interaction force curve for a typical polymer, for a fixed (*x*,*y*) point on the surface for which the force is plotted as a function of the vertical tip–sample distance and the velocity of the cantilever tip (expanded representation, left), and only as a function of the vertical tip–sample distance (traditional representation, right). All force curves shown in this paper were constructed through simulation of the spectral inversion method [11]. In all cases the origin of the vertical position (tip–sample distance) axis is located at the relaxed position of the surface. The “turning point” is the lowest vertical position reached by the tip during tip–sample impact.

In the current implementations of the spectral inversion method, the user performs a 2-dimensional (2D) scan of the surface to acquire the topography plus a tip–sample force curve, *f*(*z*)*,* at every (*x*,*y*) pixel, which effectively results in a 3D description of the tip–sample forces, *f*(*x*,*y*,*z*)*.* The force curves for each sample location are plotted as 2D graphs depicting the force as a function of the tip–surface separation, as is customary in atomic force spectroscopy (see Figure 1, traditional representation). The purpose of this paper is to introduce an extension of the method, such that the forces can be acquired in 4 dimensions (4D), as a function of the three Cartesian coordinates of the scanning volume, plus the vertical velocity of the tip (see Figure 1, expanded representation). This capability could be useful in the study of samples whose response depends on the rate of application of strains or stresses, such as polymers, composites, soft metals and biological materials, as well as in the study of other rate-dependent phenomena, such as binding or folding/unfolding events in complex biomolecules.

## Methods

The details of the spectral inversion method using the torsional harmonic cantilever have been described in detail elsewhere [8,11], so they are discussed only briefly here. In order to implement the mathematics of the force inversion procedure, it is assumed that the fundamental torsional eigenmode follows the dynamics of a damped harmonic oscillator, whose transfer function is

[1]
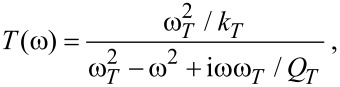


where ω is the angular frequency, ω*_T_* the torsional resonance angular frequency, *k_T_* the torsional force constant, which has been linearized in the vertical direction, and *Q_T_* is the torsional quality factor. As the cantilever base is oscillated in the vertical direction by the piezo shaker, the fundamental flexural eigenmode is excited such that the tip undergoes intermittent contact with the surface, which in turn excites the torsional eigenmode (recall that the tip is located on one of the arms of the “T” and not on the center of the cantilever end). Since the torsional eigenmode is not *directly* driven by the piezo shaker, the only driving force acting on it is the time-dependent tip–sample interaction, *f_d_*(*t*) *= F_ts_*[*z_c_*(*t*) *+ z_p_*(*t*)], which generates a torsional response that can be linearized in the *z*-direction, *z_p_*(*t*)*.* Here *F_ts_* is the tip–sample interaction force, which is a function of the distance between the tip and the sample (tip position). The tip–sample distance, in turn, is determined by the flexural cantilever position, *z_c_*(*t*)*,* plus the deflection of the torsional “paddle” (arms of the “T”) with respect to the flexural position, *z_p_*(*t*)*.* Invoking the definition of the transfer function, one can write the response of the torsional oscillator in Fourier space as

[2]
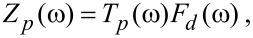


where *Z_p_*(*ω*) is the spectrum of *z_p_*(*t*)*, F_d_*(ω) is the spectrum of *f_d_*(*t*)*,* and *T_p_*(ω) is the transfer function, defined in Equation 1*.* Since it is possible to measure the deflection of the torsional oscillator, *z_p_*(*t*), in real time as the cantilever taps on the sample, one can easily calculate its spectrum, *Z_p_*(ω), through application of the fast Fourier transform to a sequence of values of *z_p_*(*t*) recorded at regular intervals. Additionally, one can also obtain the spectrum of the driving force by rewriting Equation 2 as

[3]
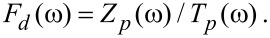


Next, one can apply the inverse fast Fourier transform to *F_d_*(ω) in order to obtain *f_d_*(*t*)*,* that is, the time-dependent tip–sample interaction force acting on the cantilever tip. Finally the force curves are obtained by plotting *f_d_*(*t*) as a function of the vertical tip position, which is generally approximated by the position of the cantilever flexural oscillation, *z_c_*(*t*) (that is, neglecting *z_p_*(*t*)), under the assumption of negligible torsional oscillation compared to the length scale over which the tip–sample forces vary.

In the ideal application of the spectral inversion method, it would be desirable to acquire *Z_p_*(ω) for the widest possible frequency range, such that all features of the force curve (e.g., sharp turns at the location of maximum attractive force) can be accurately recovered. However, this is difficult because the spectrum of the torsional eigenmode, *Z_p_*(ω), follows the general shape of the transfer function, *T_p_*(ω), which rapidly decays as the frequency deviates from the resonance frequency (recall that harmonic oscillators exhibit a Lorentzian frequency response). As a result, the high-frequency peaks in *Z_p_*(ω) become smaller and smaller as ω increases, such that their signal-to-noise ratio decreases rapidly as ω increases. Thus, in order to prevent high-frequency noise in the data from being magnified into the recovered force curves through division by very small values of *T_p_*(ω) (see Equation 3), one applies a cutoff to *Z_p_*(ω) [7–8]. This cutoff is generally set to include only a few harmonics (often only one) above the torsional resonance frequency.

In order to obtain a 4D representation of the tip–sample forces, it is necessary to also measure the tip velocity in real time, which can be easily recovered from the flexural position, *z_c_*(t) (again, neglecting *z_p_*(*t*)), by using the well-known property of Fourier analysis that states that

**Figure 2:**
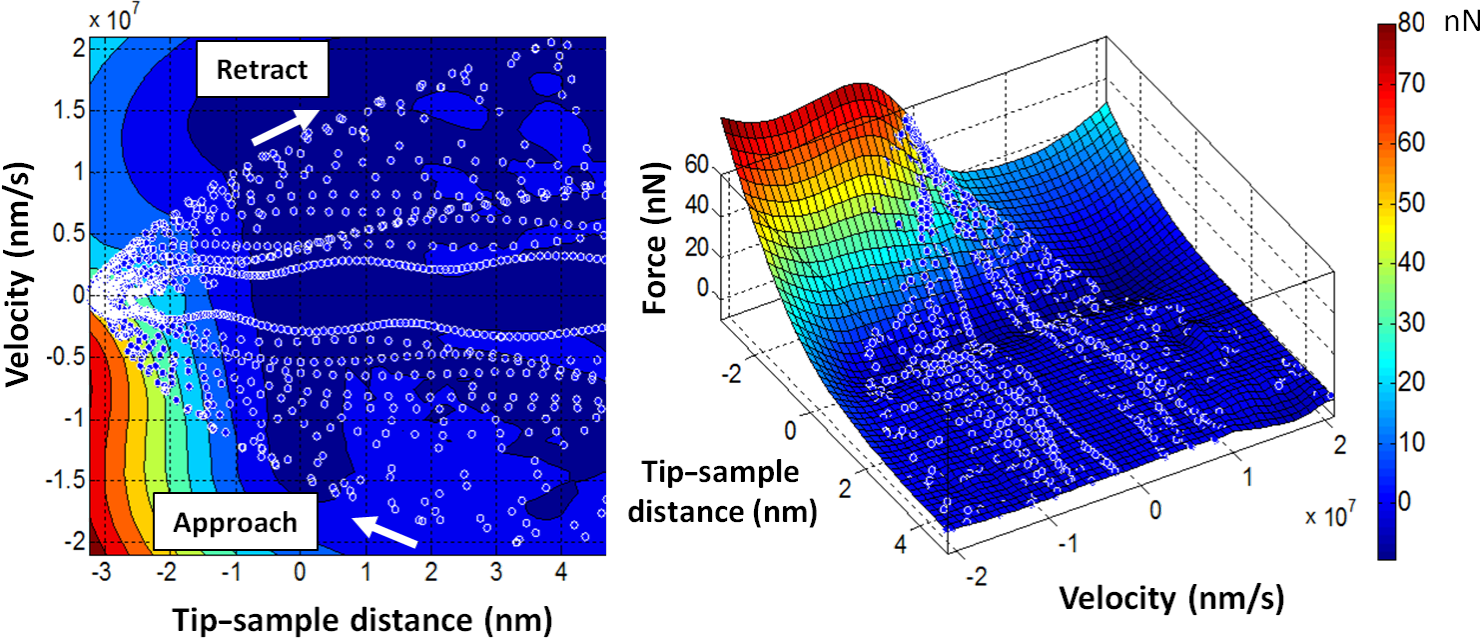
Collection of reconstructed tip–sample interaction force curves for a fixed (*x*,*y*) point on the surface, each similar to that shown in Figure 1 (expanded representation), plotted together as a plane. In this simulation, different force curves were acquired for different rest positions of the cantilever above the surface and were all plotted together. The plots show two different views of the same data. The white open circles represent 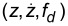 points and each force curve is a succession of such points that follows the direction indicated by the white arrows. That is, during the approach, the tip travels towards and into the surface (with negative velocity) down to the turning point, and then travels upward (with positive velocity) away from the sample. Different force curves have different incoming and outgoing velocity.

[4]
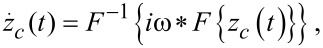


where the operators *F*{} and *F*^−1^{} are the Fourier transform and the inverse Fourier transform, respectively. Recovery of the velocity in the Fourier domain allows averaging of multiple oscillation cycles as well as filtering of the data in order to reduce the noise typically seen in the photodetector signal and in order to filter out the effect of flexural–torsional cross-talk [8,11–12]. Upon completion of the scan and post-processing of the data [11], the user will have acquired 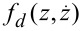 for every (*x*,*y*) pixel on the surface (see Figure 1, expanded representation), which is equivalent to the 4D representation 
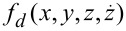
 for the full scan. It is also possible to perform spectroscopy at a fixed (*x*,*y*) position on the surface, acquiring successive force curves while varying a particular imaging parameter, such that multiple force curves following different 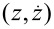 trajectories can be combined in representing the probe–sample interaction as a corrugated plane instead of a single force curve. An example is provided in Figure 2. We point out that although the proposed expanded method provides data in four dimensions, it is not an *unrestricted* 4D force-measurement method. This is because the velocity cannot be varied arbitrarily, but is instead directly related to the tip position and both are governed by the dynamics and properties of the cantilever–sample system. Thus, depending on the system, there will be regions of the 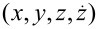 phase space for which the force cannot be measured because not all four coordinates can be varied independently of one another.

**Figure 3:**
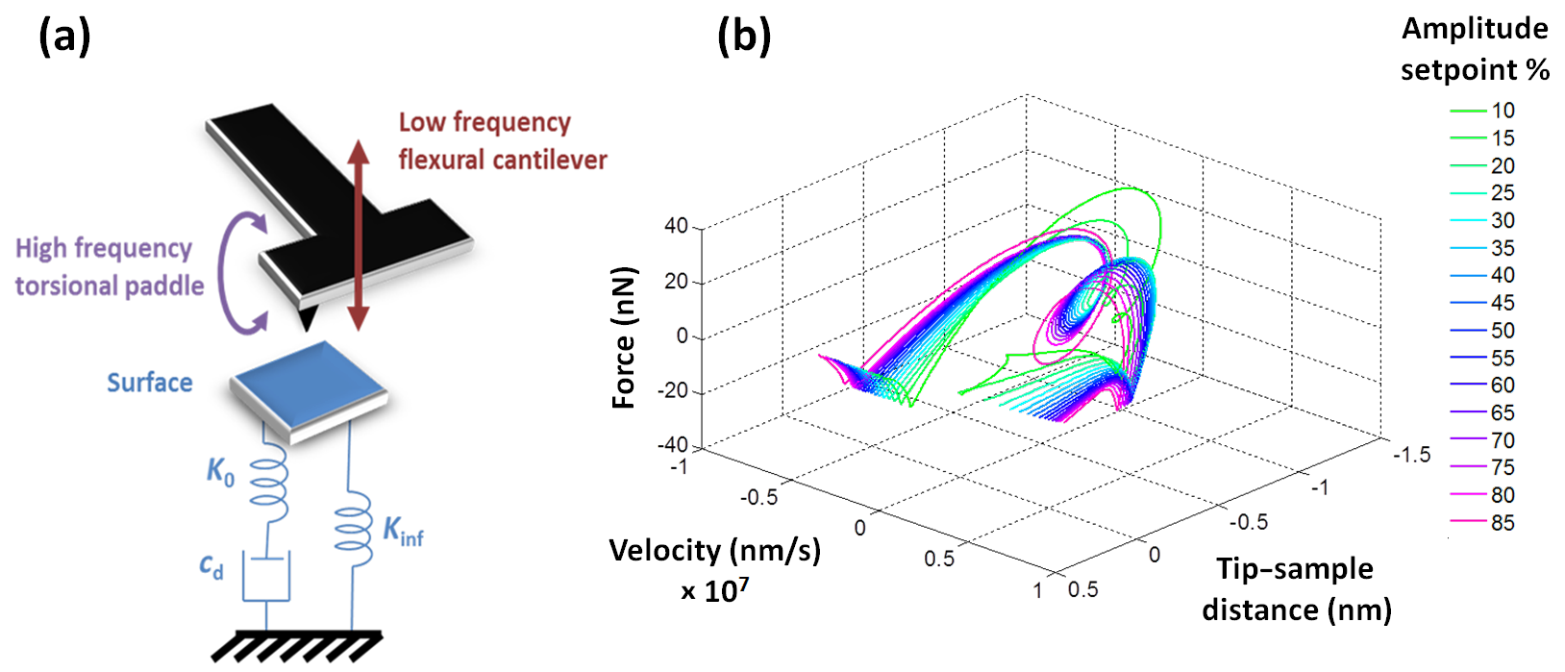
(a) Schematic of a torsional harmonic cantilever interacting with a surface modeled as a standard linear solid (*K*_o_ and *K*_inf_ represent linear springs and *c*_d_ represents a linear damper); (b) collection of force curves acquired for different values of the amplitude setpoint by using the same cantilever. Note that the force curves in (b) exhibit a loop, indicating that the tip travels into and towards the surface twice during each flexural cycle resulting in a “double tap”. This can occur whenever two eigenmodes with different natural frequencies are active. Similar phenomena take place when imaging samples in high-damping (liquid) environments [18] or in multifrequency AFM characterization [19].

The numerical integration methods and equations of motion used to simulate the spectral inversion reconstruction of the force curves presented in this paper are described in detail in [11]. Briefly, the procedure for simulating the acquisition of each individual force curve consists of (i) defining the system parameters (cantilever eigenfrequencies, force constants and quality factors, free flexural amplitude and amplitude setpoint, tip–sample force model, etc.); (ii) numerical implementation of the amplitude-modulation imaging scheme; (iii) recording of the flexural and torsional eigenmode positions as a function of time at regularly spaced intervals (digitally) for several flexural periods; and (iv) application of the inversion procedure described above to recover the tip–sample forces. The tip–sample interaction was simulated as the combination of attractive van der Waals forces (modeled through the Hamaker equation [13]) plus repulsive and dissipative interactions. In most cases, the repulsive forces were modeled by using a Hertzian contact [13], while the dissipative interactions were modeled by using a viscous force term proportional to the tip speed with a coefficient that decayed exponentially with the tip position [14] (see equations and further details in [11]). We also conducted simulations in which the conservative and dissipative interactions were accounted for through the standard linear solid model (see Figure 3 and the next section), in combination with attractive van der Waals interactions.

## Results and Discussion

### Characterization of rate-dependent phenomena

**Figure 4:**
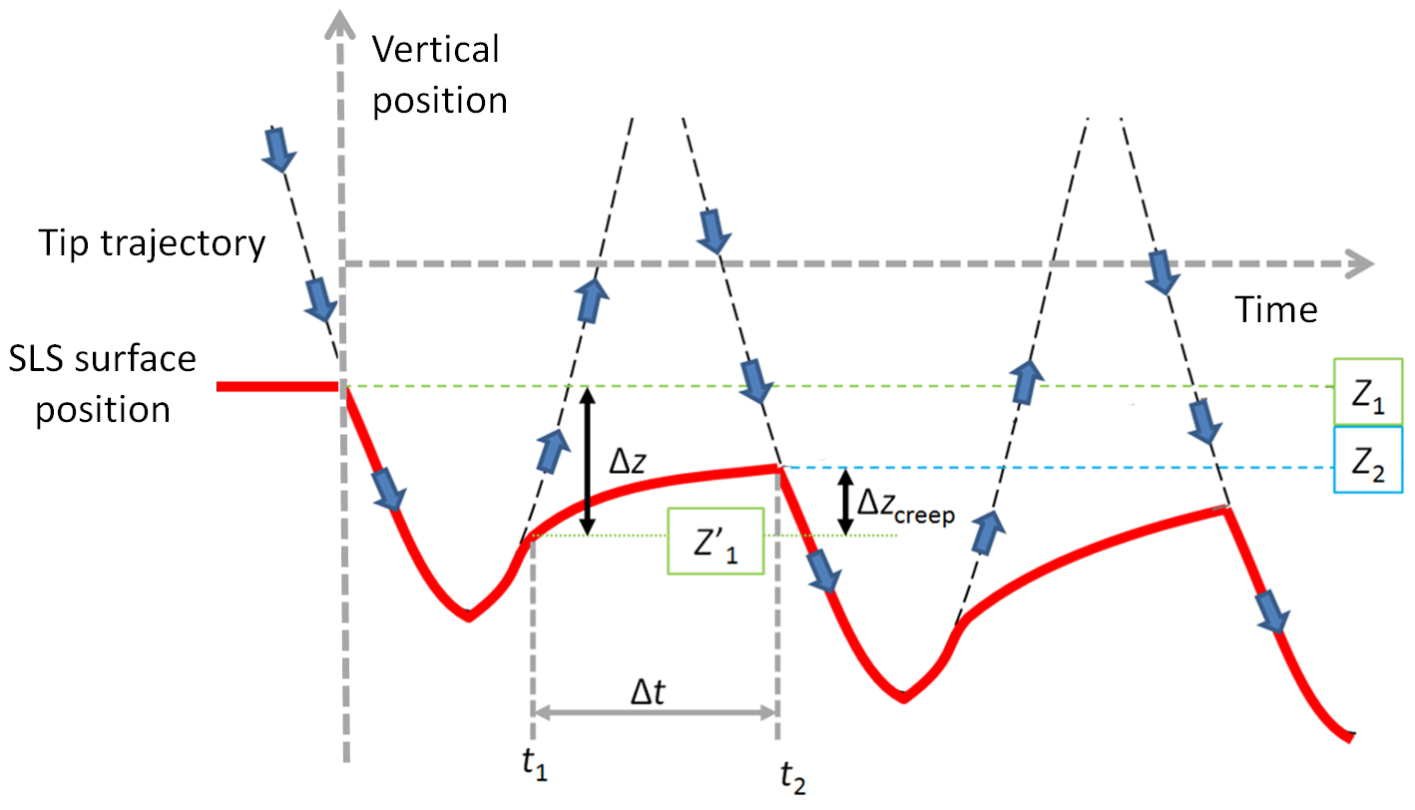
Illustration of the surface depression by the tip–sample impact, and successive recovery within the standard linear solid model. *Z*_1_ is the undisturbed surface position, before any impacts have taken place. *Z’*_1_ is the vertical tip position where tip–sample contact is lost after the first impact (loss of contact occurs because the surface recovery speed is lower than the tip speed). Between the first and second impacts the tip recovers to position *Z*_2_. If the tip continues tapping at the same surface pixel, the impact and loss-of-contact locations asymptotically approach constant values.

The ability to recover rate-dependent signatures of the tip–sample forces presents a unique opportunity to study phenomena such as plasticity, viscoelasticity and biomolecular binding and folding/unfolding. For example, it should be possible to develop methods for fitting experimental data to increasingly elaborate viscoelastic models that go beyond the Kelvin–Voigt model used in the current state of the art in contact-resonance AFM [15–16]. In particular, the Kelvin–Voigt model is not well suited to study stress relaxation. (While this paper is not intended to be a study of surface viscoelasticity, we briefly illustrate the use of slightly more elaborate surface models.) Instead, one could, for example, use the standard linear solid (SLS) model, which is a combination of the Maxwell and Kelvin–Voigt models. In the SLS configuration a Maxwell element is connected in parallel with a second spring (this setup is also known as the Zener model). The SLS approximation provides the simplest form of a linear viscoelastic approximation that can reproduce both stress relaxation and creep compliance, which are observed in the response of real viscoelastic surfaces. Figure 3a provides a schematic of our application of the 4D force mapping concept, modeling the surface as a simple SLS that also experiences van der Waals interactions with the tip. Figure 3b illustrates a “virtual experiment” in which, using the same cantilever, the user acquires multiple force curves at a single pixel by varying the amplitude setpoint. One can also conceive other types of studies in which one varies other parameters, such as the cantilever stiffness, mass, eigenfrequency ratios, etc., keeping all other parameters constant. While not all these studies are experimentally feasible, they can provide significant insight into the evolution of the 4D force representation, individual 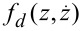 trajectories, and optimum imaging parameters for different types of systems. Due to the nature of the SLS, one standard feature of the corresponding 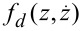 trajectories, which is evident in Figure 3b, is that the vertical tip position at which the tip impacts the surface is not the same location at which the tip leaves the surface (that is, the surface can remain temporarily indented after being impacted by the tip). In fact, depending on the horizontal scan speed of the cantilever and the number of taps that take place at every pixel, the tip may contact the surface at different heights during successive impacts, as illustrated in Figure 4. Due to the simplicity of the SLS model, analytical expressions exist for various aspects of its behavior, such as the rate of recovery of the surface (for example, the surface height recovery from position *Z’*_1_ to position *Z*_2_ between times *t*_1_ and *t*_2_ in Figure 4 can be expressed analytically). As a result, it is possible to fit the three parameters of the model from reconstructed force curves, even without explicitly acquiring velocity information, provided that the force curve inversion is accurate (a detailed analysis of parameter recovery from force curves for the SLS model can be found in [17]).

### Experimental feasibility

**Figure 5:**
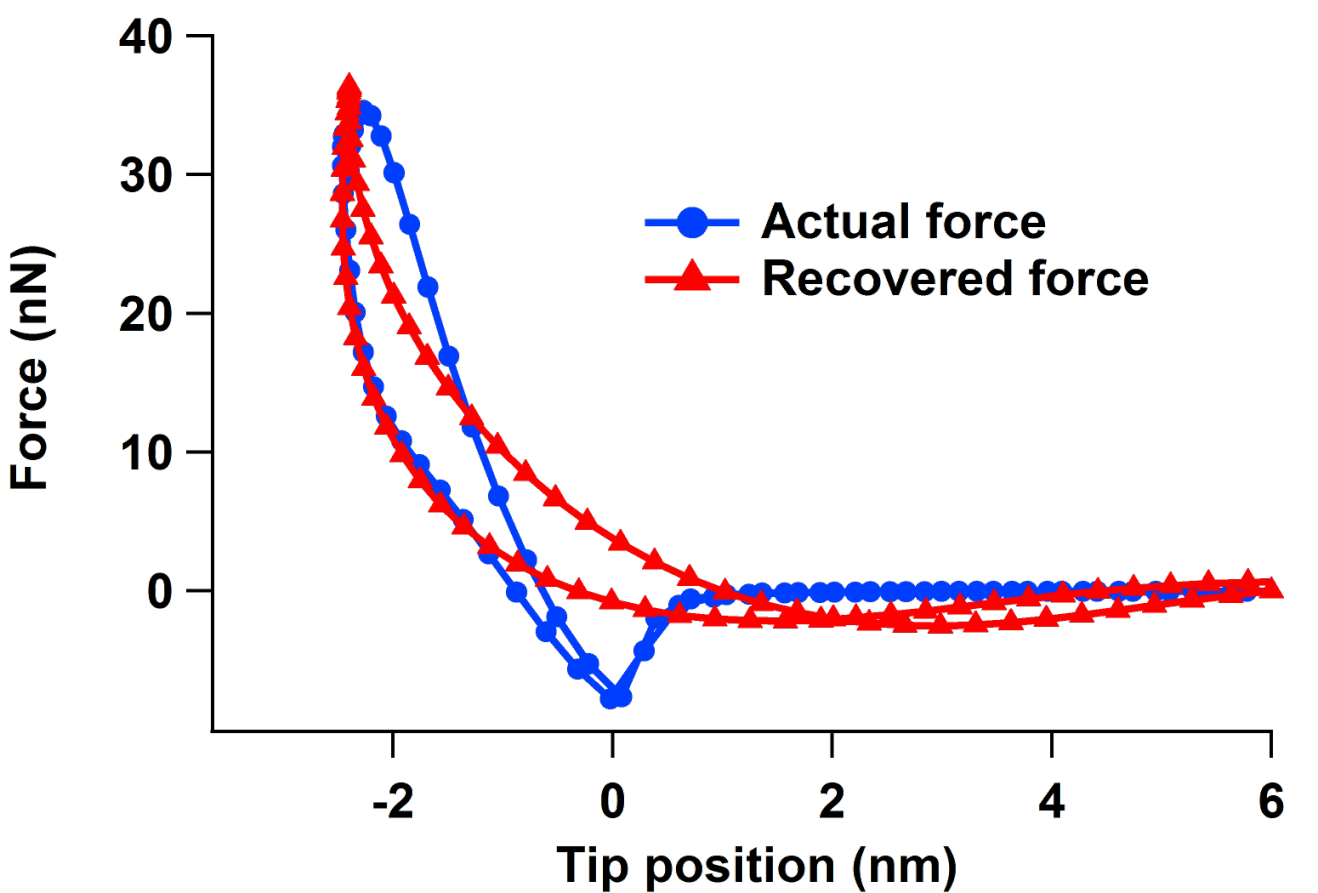
Example of force curve distortion for a case in which both conservative and dissipative interactions are present. The recovered curve uses a frequency cutoff of one harmonic above the torsional resonance frequency, similar to what is customary in experiments, and contains a very shallow and shifted minimum which differs for the approach and retract (compare to Figure 6b in [11], which shows the high-quality force curve recovered by using all available harmonics). The sample modulus of elasticity in this simulation is 2 GPa. The force curve was simulated as the sum of a conservative Hamaker–Hertzian contact with a height-dependent viscous term [14] (this combination of models does not consider surface depression or recovery, so the location of the force minima is the same for the tip approach and retract).

The additional post-processing demands required to extend the spectral inversion method from three to four dimensions are relatively minor, since the tip position data is already recorded. Furthermore, all Fourier analysis is carried out during a post-processing step and the calculation of the velocity does not represent an excessive computational burden. Thus, the 4D implementation is relatively straightforward, requiring no new technology. However, important limitations still exist in performing the method upgrade accurately and using it meaningfully, depending on the application. In particular, the study of viscoelastic models in intermittent contact AFM demands very high accuracy in order to reproduce the sharp curvatures and intricate features of the force curves [17]. While the torsional harmonic cantilever leads to an enhanced implementation of the original spectral inversion procedure, which is sufficiently accurate to estimate the effective Young’s modulus of soft samples, it does not solve the issues of signal-to-noise ratio for the *higher* frequencies in the spectrum (that is, for frequencies that are appreciably higher than the torsional eigenfrequency). This challenge becomes more significant as the sample stiffness increases (see for example, Figure 7 in [11]) and can compound itself with distortions in the force curve that may emerge in the presence of dissipation, whereby the hysteresis loops in the tip–sample force curve can change shape or shift along the tip-position axis as harmonics are removed from the spectrum (see Figure 5). Even for a simple SLS surface, it is not possible to recover the model parameters unless the impact and loss-of-contact tip positions, both of which are located at sharp minima on the force curve, can be determined accurately (see Figure 3, Figure 4 and [17]). A second important consideration concerning accuracy is that when the torsional oscillation amplitude becomes significant with respect to the length scale over which the tip–sample forces vary, accuracy is lost when the *z*-position in the force curves is approximated by *z_c_*(*t*) in place of *z_c_*(*t*) *+ z_p_*(*t*) (this is also discussed in detail in [11]). The use of the correct expression describing the time-dependent tip position is mathematically straightforward, but is not trivial in an experiment, where there can be signal cross-talk [8,11] and where calibration and noise limitations are present. For all these reasons, it is recommended to apply the method in combination with simulations [11–12], such that the user has a theoretical estimate of the errors incurred in the characterization. Finally, as already implied above, there remain unsolved signal-to-noise ratio challenges even when using the torsional harmonic cantilever, so hardware and cantilever development opportunities still exist, especially in terms of enhancing and expanding the region of the cantilever spectrum that can be recovered with a high signal-to-noise ratio.

## Conclusion

We have presented a simple, yet potentially powerful upgrade to the spectral inversion method of atomic force microscopy, which makes possible the mapping of the tip–sample interaction forces in four dimensions (the three Cartesian coordinates in the scanning volume plus the vertical velocity of the probe). We have also highlighted important limitations that still exist in the accurate experimental implementation of this procedure. Despite the unsolved challenges, the proposed approach could, in combination with future instrumentation and cantilever upgrades, enable studies in which rate-dependent phenomena, such as viscoelasticity and plasticity, are characterized in real time by using tapping-mode atomic force microscopy.
